# Expansion of Submucosal Bladder Wall Tissue* In Vitro* and* In Vivo*

**DOI:** 10.1155/2016/5415012

**Published:** 2016-09-29

**Authors:** Gisela Reinfeldt Engberg, Clara Ibel Chamorro, Agneta Nordenskjöld, Magdalena Fossum

**Affiliations:** ^1^Department of Women's and Children's Health and Center for Molecular Medicine, Karolinska Institutet, Stockholm, Sweden; ^2^Department of Pediatric Surgery, Pediatric Urology Section, Astrid Lindgren Children's Hospital, Karolinska University Hospital, Stockholm, Sweden

## Abstract

In order to develop autologous tissue engineering of the whole wall in the urinary excretory system, we studied the regenerative capacity of the muscular bladder wall. Smooth muscle cell expansion on minced detrusor muscle* in vitro* and* in vivo* with or without urothelial tissue was studied. Porcine minced detrusor muscle and urothelium were cultured* in vitro* under standard culture conditions for evaluation of the explant technique and in collagen for tissue sectioning and histology. Autografts of minced detrusor muscle with or without minced urothelium were expanded on 3D cylinder moulds by grafting into the subcutaneous fat of the pig abdominal wall. Moulds without autografts were used as controls. Tissue harvesting, mincing, and transplantation were performed as a one-step procedure. Cells from minced detrusor muscle specimens migrated and expanded* in vitro* on culture plastic and in collagen.* In vivo* studies with minced detrusor autografts demonstrated expansion and regeneration in all specimens. Minced urothelium autografts showed multilayered transitional urothelium when transplanted alone but not in cotransplantation with detrusor muscle; thus, minced bladder mucosa was not favored by cografting with minced detrusor. No regeneration of smooth muscle or epithelium was seen in controls.

## 1. Introduction

Lack of tissue can be a challenge in urological reconstructive surgery. When stomas for intermittent bladder self-catheterization or bladder augmentation are needed, autologous intestinal tissue is used primarily [1]. When intestinal tissue is incorporated into the urinary system, this comes with the risk of stone formation and an increase in mucus production, as well as urinary tract infections, and there are even reports on an increased risk of malignancy [2, 3]. Developing new biodegradable scaffolds for tissue regeneration of the wall of the urinary excretory system is therefore an important step in introducing new therapeutic treatments in the field of urological regenerative medicine. Complete autologous tissue expansion would be an appealing approach in this field.

The whole urinary excretory system basically presents with the same structural anatomy: the urothelium facing the lumen and primarily serving as a physical barrier for urine, the* lamina propria*, a layer with loose connective tissue with capillaries and nerves, and, finally, the detrusor muscle with the different layers of smooth muscle in the urinary bladder.

The ability of the urinary bladder to maintain an intact barrier, despite volume and pressure alterations, is largely dependent on features of the surface layer of the urothelium with its transitional epithelium including umbrella cells as the top layer cells facing the lumen. There is growing evidence that all the layers of the urinary bladder urothelium exhibit specialized sensory properties that play a key role in the detection and transmission of both physiological and nociceptive stimuli, including the ability to respond to chemical, mechanical, and thermal impulses that probably communicate the state of the urothelial environment to the underlying nervous and muscular systems [4]. The signaling system within the urothelium in the bladder is also considered to be of vital importance for smooth muscle differentiation [5, 6].

The signaling systems between cells in the different layers of the urinary bladder wall are not yet fully understood, but it has been shown that coculturing human smooth muscle cells and urothelial cells* in vitro* on synthetic hydrogel matrices, as well as on intestinal submucosa, creates a positive environment for proliferation of both cell types [7–9].

Epithelium has been shown to regenerate from the edge of a cut surface by migration and cell division. This was clearly described by Meek in 1958 and led to the idea that by evenly cutting a square of skin epithelium twice the regeneration capacity increased by 100% [10]. Our previous studies confirm that urothelial epithelium regenerates from the wound edges in a similar way [11, 12]. Regeneration of the bladder wall tissue beneath the urothelium has been less well described; however,* in vitro* expansion of smooth muscle cells is made possible by using explant techniques [13] and this suggests that migration to the edge of a wound before proliferation is a possible mode of action. Studies on smooth muscle cells on seeded urethral scaffolds have demonstrated the possible strengthening capacity of the urothelial structure over time [7, 13, 14].

The concept of transplanting minced tissue from the urinary system is novel. In previous studies, we demonstrated that minced particles of skin or urothelium could expand, reorganize, and form a continuous epithelium facing the luminal side four weeks after transplantation [15]. In these cases, the minced urothelium was transplanted on 3D moulds placed in the subcutaneous tissue of the abdominal wall in a porcine model. In a similar fashion, we expanded the study by transplanting the 3D mould with minced urothelial tissue particles of urothelium as a tube connecting the urinary bladder to the abdominal wall. On removing the tube, we could use the intact conduit for filling and emptying of the urinary bladder. Histological studies showed a continuous multilayered stratified epithelium of urothelial origin. The main limitation when using the conduit was the susceptibility of the neourothelium to detachment due to mechanical trauma such as catheterization.

The primary aim of the present study was to evaluate whether detrusor muscle could be expanded in a way similar to that of urothelium and, secondly, whether the detrusor muscle components could strengthen the subepithelial tissue by formation of a submucosal smooth muscle layer and thereby strengthen the attachment of the urothelium. By adding progenitor cells in the subcutaneous tissue, we hypothesized that a muscular wall could be regenerated around the neoepithelium. By these means, we aimed to improve the quality of a bladder conduit for catheterization.

## 2. Materials and Methods

The protocol was preapproved by the Stockholm County Committee on Animals and all the procedures conformed to local regulations for use of animals, as well as relevant national statutes.

### 2.1. Surgical Procedures

Nine 15–25 kg (11-12 weeks old) female Yorkshire-Swedish Country Pigs (Vallrum) were used for the* in vivo* and* in vitro* studies. After 12-hour fasting, an intramuscular injection of azaperone 2 mg/kg (Stresnil, Janssen-Cilag, Pharma) was given as premedication prior to the injection of a combination of tiletamine hypochloride 2.5 mg/kg, zolazepam hypochloride 2.5 mg/kg (Zoletil, Virbac), medetomidine 25 *μ*g/kg (Dormitor, Orion Pharma), and atropine 25 *μ*g/kg (Atropin, Mylan Inc.) for induction of anaesthesia. Buprenorphine 45 *μ*g/kg (Temgesic, RB Pharmaceuticals) and carprofen 3 mg/kg (Rimadyl, Orion Pharma) were administered intravenously preoperatively for analgesia. A local injection of lidocaine (Xylocaine, AstraZeneca) was used prior to skin incision.

Phenobarbiturate 15 mg/kg (Pentobarbital, APL) was administered before endotracheal intubation and general anaesthesia was maintained with 0.8–2% isoflurane (Isoflurane, Baxter). Glucose 25 mg/mL (Baxter) was administered intraoperatively and blood pressure, pulse, saturation, and body temperature were monitored during the whole procedure. Paraffinum liquidum (Oculentum simplex, APL) was used for eye protection.

The abdominal skin was cleansed with chlorhexidine gluconate (Hibiscrub 40 mg/mL, Regent Medical) and with chlorhexidine (Klorhexidin 5%, Fresenius Kabi).

The urinary bladder was catheterized with an 8 Fr latex silicone catheter, emptied, and then refilled with 37°C sterile saline solution (NaCl, Sigma-Aldrich) (approximately 8 mL/kg body weight).

With the pig in a supine position, a midline incision below the umbilicus was performed in order to expose the urinary bladder. A lenticular area of the urinary bladder dome, measuring 12 cm longitudinally by 9 cm transversally (approximately 1/4 of the total urinary bladder surface area), was marked and removed. The urinary bladder was then closed with double layers of continuous 5-0 and 4-0 absorbable monofilament sutures (Biosyn, Covidien). The abdominal fascia was closed with abdominal braided running 3-0 sutures and the subcutaneous tissue with 4-0 sutures (Polysorb, Tyco Healthcare). The skin was closed with interrupted 3-0 nylon sutures (Ethilon, Ethicon, Johnson & Johnson, Sweden).

**Figure 1:**
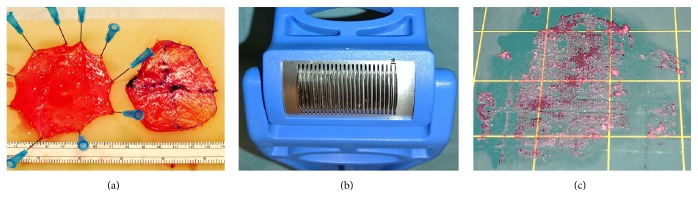
(a) Preparation of minced tissue. Pinned bladder mucosa on the left and detrusor muscle, with markings from measurements on the right, after mechanical separation. (b) Skin grafting instrument demonstrating the rotating cutting discs 0.8 mm apart marked with (*∗*). (c) Minced urinary bladder tissue.

The excised urinary bladder tissue was washed twice in Dulbecco's modified Eagle's medium (Sigma-Aldrich) and the detrusor muscle was separated mechanically from the bladder mucosa (Figure 1(a)). The thin peritoneal membrane was then removed from the detrusor with surgical scissors and the detrusor muscle was minced with a mincing device developed for mincing of skin (XPANSION® Skin Grafting Instruments, Applied Tissue Technologies LLC) as shown in Figure 1(b). The mincing device cut the tissue with 30 rotating sharp discs placed parallel to each other at a distance of 0.8 mm. By running the device twice on the tissue, in perpendicular directions, particles measuring approximately 0.8 × 0.8 mm were formed. The same procedure was undertaken with the bladder mucosa (Figure 1(c)).

### 2.2. *In Vitro* Studies

#### 2.2.1. Cell Expansion in Culture Plastic

Minced detrusor muscle tissue particles were placed in 24-well cell culture plastic and placed in a 37°C incubator with smooth muscle cell culture media (DMEM and Ham's F12 (4 : 1 mixture; Gibco), foetal bovine serum (10%, Gibco), and antibiotics (penicillin 50 U/mL and streptomycin 50 *μ*g/mL)) for cell expansion* in vitro*. Minced urothelial tissue particles were handled in a similar way but were cultured in epithelial cell culture media (DMEM and Ham's F12 (4 : 1 mixture; Gibco), foetal bovine serum (10%, Gibco)), insulin (5 *μ*g/mL), hydrocortisone (0.4 *μ*g/mL; Calbiochem VWR, 386698), adenine (21 *μ*g/mL; Sigma), cholera toxin (10^−10^ mol/L; Sigma C3012), triiodothyronine (2 × 10^−9^ mol/L), and antibiotics (penicillin 50 U/mL and streptomycin 50 *μ*g/mL). The medium was changed every other day.

#### 2.2.2. 3D Cell Expansion on Collagen Scaffolds

In order to study cell expansion in a 3D model that could be handled mechanically for tissue sectioning and histology, we used a cell-scaffold hybrid construct as previously described [16–18]. Briefly, a collagen gel was prepared from rat-tail collagen type I (First Link Ltd.) by mixing with 10x Eagle's minimum essential medium (MEM; Invitrogen), neutralizing with NaOH, and then adding Dulbecco's modified Eagle's medium (DMEM; Gibco). The gel was then cast in a rectangular mould and incubated at 37°C for 10 min. A PCL-knitted (polycaprolactone-knitted) mesh was placed onto the gel and remaining collagen solution was cast into the same mould to form a second layer of collagen which was then incubated for 20 min. Minced tissue was seeded on top of the scaffold and plastic compression was performed at room temperature for 5 min using a weight of 120 g on top of the mould.

**Figure 2:**
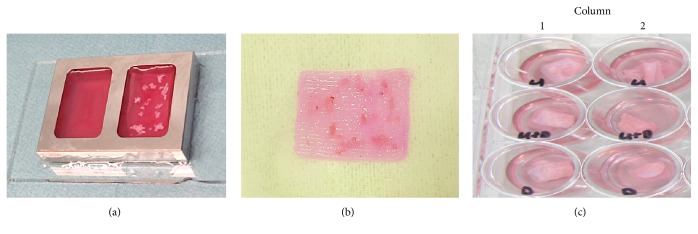
(a) Preparation of 3D collagen scaffolds* in vitro*. Minced tissue placed on top of the collagen gel on the right, before plastic compression. Collagen without minced tissue on the left. (b) Scaffold containing minced bladder tissue after plastic compression. (c) Scaffolds containing minced tissue cut to fit in 12-well plates in cell culture. Columns 1 and 2 demonstrating duplicates for urothelium (U), urothelium and detrusor (U + D), and detrusor (D).

The scaffolds' upper surface was covered with minced tissue consisting of detrusor only, detrusor and urothelium, or urothelium only which was evenly and randomly spread, aiming for an overall 1 : 3 expansion (Figure 2(a)).

Scaffolds containing minced tissue were cut to fit in 12-well plates, as shown in Figure 2(b), placed in epithelial cell culture medium, and cultured in duplicates (Figure 2(c)). The wells were maintained in 5% carbon dioxide and humidified air at 37°C atmospheric pressure. The medium was changed every other day and samples were cultured in duplicates and analyzed after two or three weeks.

### 2.3. *In Vivo* Studies

The bladder tissue was obtained with the bladder mucosa and the detrusor muscle minced separately and as previously described. The appropriate amount of minced autologous tissue was calculated by measuring the outer surface of a size 16 Fr 2 cm long (16 Fr × length) cylinder made of latex and covered with a silicon elastomer (Bard Foley Catheter, C.A. Bard Inc.), sealed with 4-0 nylon sutures (Ethilon, Ethicon, Johnson & Johnson) at each end, and dividing by three, aiming for a 3-fold expansion around the mould for every specimen of minced tissue. Thus, moulds with detrusor and urothelium carried twice as much tissue as the other moulds with detrusor or urothelium only.

The outer surface of the mould was covered with two thin layers of the sealer protein concentrate (fibrinogen) from a two-component tissue sealant (Tisseel VH, Baxter). The thrombin component was added immediately after the second application of fibrinogen, and pieces of minced tissue were applied evenly and randomly distributed. Three of the pigs also received transplants of moulds covered with the two-component tissue sealant but without minced particles.

**Figure 3:**
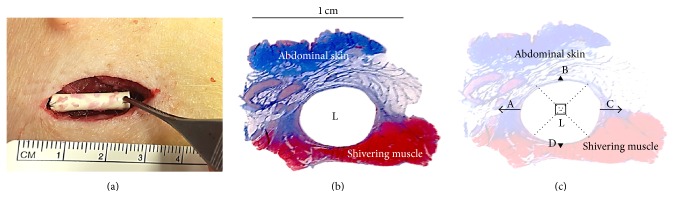
(a)* In vivo* porcine model. Tweezers holding a 2 cm long mould of 16 Fr catheter with minced tissue particles. Skin incision in the abdominal wall down to the shivering muscle. Photograph of a microscopic slide with tissue from a cross section demonstrating the formation of a tunnel after removal of the mould. (b) (Masson's trichrome). (c) Washed-out photograph of a microscopic slide with tissue from a cross section demonstrating scoring of generated granulation tissue and number of capillaries in four different directions 90° apart, indicated by the arrows and the dotted cross section, with L indicating the lumen. The sides are marked A, B, C, and D, corresponding to the four locations in Table 1 (Masson's trichrome).

Transcutaneous incisions lateral to the nipple ridge were performed down to the shivering muscle (*panniculus carnosus*). Diathermy was used for haemostasis and a subcutaneous space was created for horizontal placement of the cylindrical mould with the minced autologous tissue in the subcutaneous fat of the abdominal wall (Figure 3(a)). The edges of the mould were secured to the underlying tissue with 4-0 nylon sutures (Ethilon, Ethicon, Johnson & Johnson) and the subcutaneous fat was closed with continuous 3-0 and 4-0 absorbable monofilament sutures (Biosyn, Covidien) to create a tight-fitting pocket in order to minimize movement. The skin was closed with interrupted 3-0 nylon sutures (Ethilon, Ethicon, Johnson & Johnson).

One to six moulds were used in each animal. A total of 23 moulds were transplanted and distributed as follows: autologous minced detrusor (6), autologous minced detrusor and urothelium (7), autologous minced urothelium (7), and shams without any minced tissue (3) serving as controls. The type of transplant on each 3D mould or in the sham operation was randomly selected before the operation on the pig.

*In vivo* specimens of subcutaneous tissue including the 3D mould were surgically removed between four and five weeks (Figure 3(a)).

### 2.4. Postoperative Procedures

Protocols concerning the animals' well-being included controls for urination preoperatively and postoperatively, signs of infections, surgical side effects, and check-ups on dressings and wounds several times daily throughout the study period. All animals tolerated the excision and removal of bladder tissue well and voided shortly after surgery. None of them developed postoperative fever or signs of strain or pain when voiding. One pig was euthanized on the first postoperative day owing to signs of pain. The autopsy showed a perforated ulcer of the ventricle. This resulted in eight animals surviving the planned study period.

The pigs had a full supply of fresh water and were fed according to standard protocols at the animal institution where they were stalled. Animals were kept in pairs, except immediately postoperatively, until they regained normal activity. After removal of the tissue transplants under general anaesthesia, the animals were euthanized with a lethal dose of sodium thiopental (Sodium Pentothal, Abbot Laboratories).

### 2.5. Histological Analysis

#### 2.5.1. Preparation and Staining

*In vitro* tissue specimens of collagen scaffolds and* in vivo* transplant specimens were fixed in 10% formalin solution prior to dehydration. Paraffin embedding and slicing into 5 *μ*m thin sections were carried out with all specimens. The* in vivo* specimens were cut transversally into halves and the piece of mould was taken out with tweezers prior to embedding and slicing. Histological analyses were obtained at up to three different sites on the* in vivo* specimens, excluding 5 mm of each end.

Slides were stained with haematoxylin-eosin (H&E) for routine histology and Masson's trichrome for distinguishing newly regenerated tissue (granulation tissue) from surrounding native connective tissue.

The tissue specimens were further evaluated by immunostaining with primary antibodies against various cytokeratins (1 : 300) (pancytokeratins 1–7, 10, 13–16, and 19, DakoCytomation, Cat#M0821, clone: MNF 116), uroplakin III (1 : 400) (Fitzgerald Industries, Clone AU1, CatRDI-PRO610108), alpha-actin (1 : 10 000) (*α* Smooth Muscle, Sigma-Aldrich, clone 1A4, purified mouse immunoglobulin, product number A 5228), and Ki-67 (1 : 200) (Ki-67; Lab Vision, Rabbit Monoclonal, clone SP6). Biotinylated secondary antibodies were detected using the ABC-HRP kit (Vectastain Elite ABC kit, Vector Labs) and developed with 3.3′-diaminobenzidine (DAB) substrate (Sigma Chemical Co.) according to the manufacturer's instructions. Mayer's haematoxylin solution was used for counterstaining.

Histological measurements were performed under a light microscope (Axioscope 2 MOT Zeiss) with a built-in measuring device.

### 2.6. Definition of Scoring of Granulation Tissue and Capillaries

Detection of regenerated detrusor muscle was scored positive when smooth muscle was found to be clearly separated from the shivering muscle. Neoepithelialization was scored positive when facing the lumen.

The amount of granulation tissue (length in *μ*m) was measured in four locations, 90° apart (Figure 3(c)). The four different areas were specified as A (side), B (towards the abdominal skin), C (side opposite to A), and D (facing the shivering muscle) (Figure 3(c) and Table 1).

The number (*n*) of capillaries was estimated by counting those in the same different four location areas as previously described (Figure 3(c) and Table 1).

## 3. Results

### 3.1. *In Vitro*

**Figure 4:**
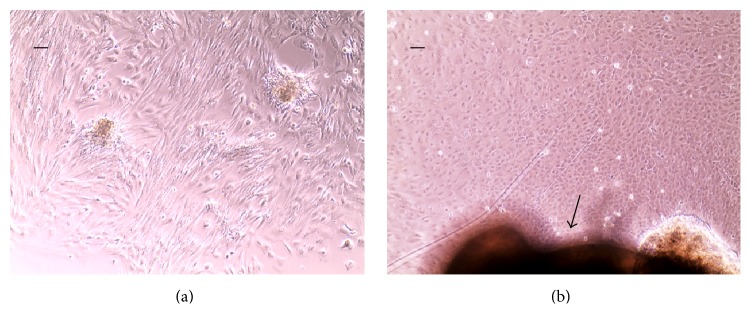
Morphology after two weeks in culture of minced detrusor and minced urothelium with explant technique. Photomicrographs of cell expansion demonstrating smooth muscle cell expansion (a) and urothelial cell expansion (b). Arrow indicating a minced particle of urothelial mucosa.

Cell expansion on culture plastic after two weeks showed cell migration and regeneration from minced tissue specimens with a morphology typical of smooth muscle cells from the minced detrusor muscle explants and urothelial cells from minced urothelium explants, respectively (Figures 4(a) and 4(b)).

**Figure 5:**
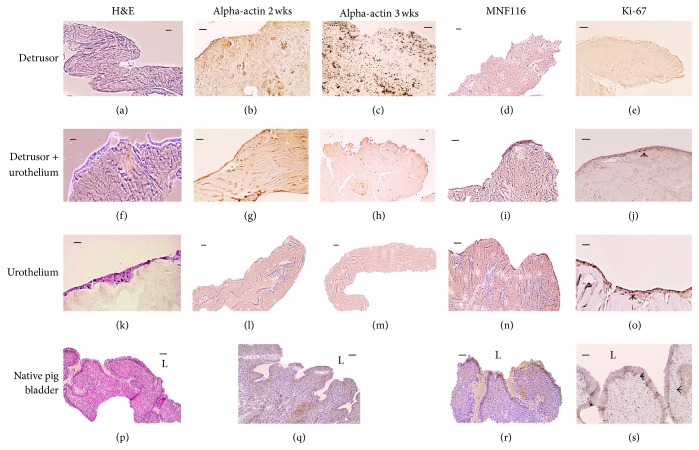
Morphology of 3D cell expansion on collagen scaffolds after two to three weeks* in vitro* culture in plastic wells; minced detrusor ((a)–(e)), detrusor and urothelium ((f)–(j)), and urothelium ((k)–(o)), compared to native pig bladder ((p)–(s)). Photomicrographs demonstrating routine staining with haematoxylin-eosin and immunostaining with alpha-actin after two and three weeks, respectively, for cytokeratin (MNF116) and Ki-67. Arrows (j, o, and s) indicating cells with a sustained proliferative capacity (Ki-67).

After two weeks, 3D cell expansion in collagen cells of nonepithelial origin could be found inside the collagen scaffolds only in cultures that had been created with minced detrusor muscle biografts (Figures 5(b) and 5(g)). Immunoassays after three weeks in culture confirmed that these were smooth muscle cells since they stained for alpha-actin (Figure 5(c)). No cells could be detected inside the collagen in cell scaffolds with urothelium only (Figures 5(k)–5(m)).

Scaffolds with minced urothelium, alone or together with minced detrusor muscle, showed epithelial lining on the collagen surface after two weeks. Immunoassay with cytokeratins confirmed the urothelial origin (Figures 5(i) and 5(n)).

The urothelium stained for Ki-67, indicating a sustained proliferation capacity (Figures 5(j) and 5(o)).

### 3.2. *In Vivo*

A total of 20 moulds were obtained for histological measurements at the planned study end-point four to five weeks after transplantation. There were five moulds with transplanted autologous minced detrusor, six moulds with minced autologous urothelium and detrusor, seven moulds with minced autologous urothelium, and two shams. Histological findings were also obtained for the pig euthanized early on: one mould of minced autologous detrusor, one mould of minced autologous detrusor and urothelium, and one sham mould serving as a control. Histology demonstrated a large amount of diffuse granulation tissue in the specimen with cotransplantation of minced autologous detrusor and urothelium. As for the specimen with only minced autologous detrusor, the amount of granulation tissue in the four different previously specified locations was equivalent to that of samples at four and five weeks after transplantation.

### 3.3. Detrusor Regeneration and Proliferation

**Figure 6:**
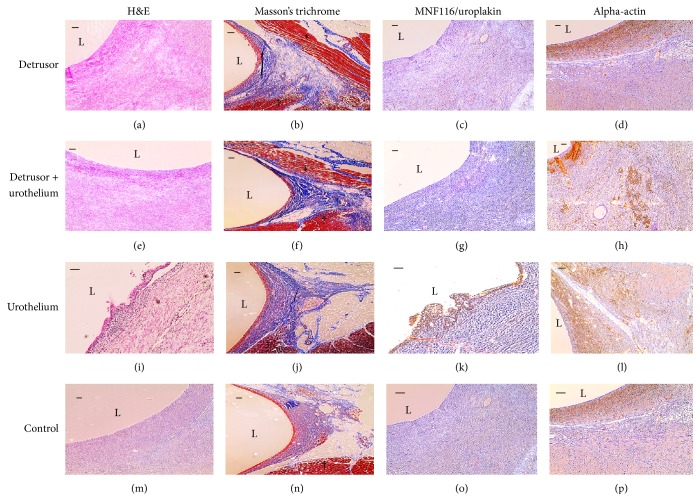
Morphology after four weeks* in vivo*. Transplants with minced detrusor ((a)–(d)), detrusor and urothelium ((e)–(h)), urothelium ((i)–(l)), and control ((m)–(p)). Photomicrographs demonstrating routine histology with haematoxylin-eosin and Masson's trichrome and immunostaining for cytokeratin (MNF116) ((c), (g), and (o)), uroplakin III (k), or alpha-actin, with L indicating the lumen. Regenerated detrusor muscle indicated by (*∗*), separated from the shivering muscle marked with (†) ((b) and (f)). (j) No regenerated detrusor muscle was shown when transplanting with only minced urothelium. (k) A multilayered continuous transitional epithelium of urothelial origin was confirmed by uroplakin in specimens with urothelium. Immunostaining with alpha-actin demonstrated smooth muscle cells in specimens transplanted with minced detrusor muscle (d) as well as minced detrusor and urothelium (h).

Data from histology analyses are shown in Table 1. The detrusor muscle reorganized and proliferated in two out of five (40%) of the transplants with minced autologous detrusor only and four out of six (67%) of the transplants with a minced autologous detrusor and urothelium (Figures 6(b) and 6(f)). The specimens showing detrusor muscle proliferation, altogether six out of 11 (55%), showed continuous muscle tissue separated from the shivering muscle and reorientation around the mould. Detrusor muscle was present neither in transplanted minced autologous urothelium only nor in shams (Figure 6(j)).

### 3.4. Urothelial Regeneration and Proliferation

A continuous transitional cell epithelium covering the luminal surface and up to five cell layers thick (Figure 6(i)) was present in transplants with minced autologous urothelium only but not in any of the moulds with cotransplanted minced autologous urothelium and detrusor muscle. The area with epithelium varied between samples and showed signs of loose attachment. As expected, no epithelial lining was detected in the six moulds transplanted with minced autologous detrusor muscle only or in shams. Immunostaining with uroplakin III confirmed the presence of urothelium (Figure 6(k)).

### 3.5. Estimation of Granulation Tissue

Calculations on granulation tissue are presented in Table 1. Submucosal tissue revealed signs of inflammation with infiltration of mononucleated cells and a less mature extracellular matrix around the moulds in all specimens compared to the matrix from bladder wall biopsy specimens taken from the same animals (Figure 6(l)). The amount of granulation tissue varied throughout the lumen, with the highest amount of granulation tissue found on the sides (A and C) of the moulds. The shivering muscle showed a natural boundary to granulation (D). Less granulation tissue was also noted towards the abdominal skin in the fatty tissue (B). All of the transplants, regardless of the type of transplanted minced tissue, showed the same distribution of granulation tissue as in controls.

### 3.6. Estimation of Capillaries

The number of capillaries, as presented in Table 1, was observed and estimated in the same specified areas as for the estimation of granulation tissue. Areas close to the shivering muscle (D) or fatty tissue facing the abdominal skin (B) showed less or no proliferation of capillaries and the areas on the side of the lumen with more granulation tissue showed a higher number of capillaries (Figure 3(c)).

## 4. Discussion

We have studied cell regeneration from minced detrusor muscle cultured* in vitro* under standard culture conditions, as well as in a 3D culture model in a collagen scaffold, with successful results with respect to cell expansion and regeneration. We then added minced detrusor muscle in an* in vivo* porcine model according to techniques we had previously developed. Our* in vivo* findings with minced detrusor tissue were the following: the minced detrusor reorganized and proliferated around the mould but did not show any beneficial effects on the urothelium. On the contrary, urothelium did not regenerate at all when detrusor muscle was added to the moulds as opposed to the urothelial lining facing the mould that was found when minced urothelium only was transplanted.

In our previous porcine minced urothelial tissue study, we demonstrated a one-stage surgical technique for creation of bladder conduits which is easy to perform and could be accessible in any standard surgical unit [15]. The rationale for adding detrusor muscle to the technique was to further improve the quality and strength of the supporting submucosal tissue and the attachment of the urothelium.

The* in vitro* studies supported the hypothesis. The reason for this not happening in the* in vivo* environment can only be speculated on; one reason could be an unbalanced relationship not only regarding the regenerative capacity of the different minced transplants with respect to the amount of regenerative cells but also regarding need for vascular supply during take of transplants. Whether this is problematic remains to be established; urothelial coverage may be achieved by outgrowth of cells from the anastomosis site.

Using the subject's own body as a bioreactor for transplanting minced tissue for epithelial expansion has been discussed in previous articles [11, 12, 15, 19]. Techniques that use these* in vivo* principals for tissue expansion could constitute major improvement in future surgical reconstructions with a lack of tissue. Advantages with the porcine model include a good-sized bladder for tissue harvesting and good tolerance to reduction of its size.

Standard processes for* ex vivo* proliferation and expansion of cells for transplantation to patients are strictly regulated to ensure safety. Studies on long-term* in vitro* cultivations of urothelial tissue indicate that urothelial cells may lose their regenerative capacity characteristics and may enter senescence after long-term culture [20, 21]. In addition, the techniques for cell culturing demand high-standard laboratory facilities that may be located far away from the patient and often incurring high costs.

Lower proliferation of smooth muscle and urothelial cells within cell-seeded scaffolds has been demonstrated when cocultured in a mixed manner rather than with layered and sandwiched cocultures [7, 22]. The reason for this is not fully understood. Our study using the mincing technique is in accordance with the mixed manner. However, the fact that the minced urothelium cells did not proliferate when transplanted with the minced detrusor did not seem to have any adverse effect on the proliferation of the detrusor.

Other studies [13, 14] have shown that when cocultured on scaffolds in the urethra the urothelium and especially the smooth muscle cells develop over time from one month and onward. Since termination was after four to five weeks in our study, we might have seen better results over time. The fact that no urothelium could be detected in the cotransplanted specimens might suggest early cell interaction in favor of muscle cells over urothelial cells when cotransplanting minced urothelium and detrusor muscle outside the urothelial region. Since transplants with only minced urothelium did develop a multilayered urothelium under the same conditions, this implies that necessary factors do exist for urothelial regeneration and expansion when transplanted outside the urothelial region.

In the* in vitro* model, the collagen acted as a substitute for the* in vivo* surrounding subcutaneous tissue in order to create a 3D environment. This technique allowed for tissue handling, sectioning, and histology. We do not know if a collagen composition would support randomly placed tissue grafts* in vivo* as this was not part of our original hypothesis. The method for inserting the minced particles in a tubular collagen shape for* in vivo* purposes has not been developed yet but might be of interest with regard to the results from our present experiments.

Further improvements in the healing conditions, such as minimizing pressure and shear stress, could result in better regeneration. In a human setting, one of the advantages after transplantation would be the supine resting position and the absence of an immediate change in body weight, such as seen in the porcine model. By these means, the pressure and the shear force on the transplant would be avoided.

In future studies, we will evaluate the construction and components of the scaffold with regard to the proliferation of different cells and coculturing of cells. The next step will be to construct a 3D cylinder mould for* in vitro* and then, eventually,* in vivo* studies to further the construction of a conduit to the urinary bladder for self-catheterization. We will also do further studies on cell proliferation and expression of markers for minced bladder tissue.

## 5. Conclusions

In conclusion, we demonstrated that minced detrusor muscle had high capacity to regenerate and reorganize, in analogy with previous studies on urothelial cells. Cell expansion was successful* in vitro* by the explant technique, as well as in collagen scaffolds.* In vivo* studies demonstrated proliferation of minced detrusor muscle in analogy with previous studies on minced urothelium. However, coculturing of minced urothelium and detrusor muscle cells did not strengthen the submucosal tissue and was not favorable for urothelial cell regeneration.

## Figures and Tables

**Table 1:** Morphological data on *in vivo* transplanted moulds.

	Detrusor	Detrusor + urothelium	Urothelium	Control
	*n* ^†^ = 5	*n* ^†^ = 6	*n* ^†^ = 7	*n* ^†^ = 2
*Granulation tissue* ^*∗*^				
A (side)	**410** (270–580)	**470 **(330–640)	**530** (280–900)	**470** (360–520)
C (side)	**470 **(280–830)	**440** (330–600)	**380** (100–720)	**460 **(400–500)
B (up)	**90** (50–150)	**105 **(70–240)	**130** (80–250)	**120** (70–190)
D (down)	**80** (30–260)	**80** (40–130)	**100** (30–200)	**60** (20–100)

*Capillaries* ^*∗∗*^				
A (side)	**12** (5–22)	**21** (12–42)	**8 **(4–13)	**11** (8–22)
C (side)	**11** (4–13)	**16 **(9–21)	**6** (1–10)	**8 **(4–12)
B (up)	**4** (0–14)	**8** (1–18)	**2 **(0–8)	**2 **(0–4)
D (down)	0 (0-0)	0 (0-0)	**2** (0–8)	1 (1-1)

^†^Number of moulds transplanted with each specific minced autologous tissue. ^*∗*^Granulation tissue measured in *μ*m. Mean values in bold followed by range of measures. ^*∗∗*^Number of capillaries observed in specified areas. Mean values in bold followed by range measures.
